# Follow-up of parenchymal changes in the thyroid gland with diffuse autoimmune thyroiditis in children prior to the development of papillary thyroid carcinoma

**DOI:** 10.1007/s40618-018-0909-x

**Published:** 2018-06-05

**Authors:** D. Januś, M. Wójcik, A. Taczanowska, P. Sołtysiak, A. Wędrychowicz, D. Roztoczyńska, G. Drabik, Ł. Wyrobek, J. B. Starzyk

**Affiliations:** 1 Department of Pediatric and Adolescent Endocrinology, Chair of Pediatrics, Institute of Pediatrics, Jagiellonian University Medical College, Wielicka St. 265, 30-663 Krakow, Poland; 2 Department of Pediatric and Adolescent Endocrinology, University Children Hospital, Krakow, Poland; 3 Department of Pediatric Surgery, University Children Hospital, Krakow, Poland; 4 Department of Pediatric Surgery, Institute of Pediatrics, Jagiellonian University Medical College, Krakow, Poland; 5 Department of Clinical Immunology and Transplantation, Institute of Paediatrics, Jagiellonian University Medical College, Krakow, Poland; 6 Department of Radiology, University Children Hospital, Krakow, Poland

**Keywords:** Autoimmune thyroiditis, Papillary thyroid carcinoma, Ultrasonography of thyroid gland, Normoechogenic background of thyroid gland

## Abstract

**Purpose:**

To present the outcomes of ultrasound (US) follow-ups in children with autoimmune thyroid disease who did not have a thyroid nodule on admission but developed papillary thyroid carcinoma (PTC) and to characterize the parenchymal changes in the thyroid gland prior to the development of PTC.

**Methods:**

A retrospective thyroid US scan review of 327 patients diagnosed with AIT was performed. Forty patients (40/327, 12.2%) presented nodular AIT variant with a normoechogenic background. Eleven patients (11/327, 3.4%, 11/40, 27.5%) presenting this variant were diagnosed with PTC (nine females—mean age 15.3 years; two males aged 11 and 13 years). In five of 11 patients, the suspicious nodule that was later confirmed to be PTC was detected on the initial US at presentation. For the remaining six females (6/11) who developed PTC during the follow-up, we retrospectively analysed their US thyroid scans and these patients were selected for analysis in this study.

**Results:**

On admission, the US evaluation revealed an enlarged normoechogenic thyroid gland in three patients and a hypoechogenic thyroid gland with fibrosis as indicated by irregular, chaotic hyperechogenic layers in three patients. No thyroid nodules were identified. Ultrasound monitoring revealed increasing echogenicity of the thyroid parenchyma during the follow-up. PTC developed in a mean time of 4.6 years (1 9/12–7 4/12 years) since referral to the outpatient thyroid clinic and 2.9 years (6/12–6 9/12) since the last nodule-free US thyroid scan.

**Conclusions:**

Sonographic follow-up assessments warrant further exploration as a strategy to determine PTC susceptibility in the paediatric population.

## Introduction

Differentiated thyroid carcinoma (DTC) accounts for the vast majority of paediatric thyroid cancers [1]. Papillary thyroid carcinoma (PTC) accounts for more than 90% of all childhood DTC cases [2]. Thyroid cancer is the most common paediatric endocrine malignancy in the United States [1]. The most recent statistical data estimate a thyroid cancer rate of 0.59 per 100,000 patients less than 19 years of age, with the incidence increasing at a rate of approximately 1% per year [3]. The annual incidence of thyroid cancer is < 1 case per million/year in children under 10 years of age, 3.5 cases per million/year in children between the ages of 10 and 14 years, and 15.4 cases per million/year in those aged 15–19 years [4–8]. According to the data of the Polish National Cancer Registry from 2003 to 2013, new cases of thyroid cancer in patients less than 19 years of age constitute every second solid neoplasm in girls and every eighth solid neoplasm in boys [2].

The prevalence of chronic autoimmune thyroiditis (AIT) is reportedly up to 2% in children and almost 10% in adolescents and continues to rise [9, 10]. The disease has a female predominance, and although observed in children up to 3 years of age, it often occurs after the age of six and peaks at 10–12 years of age [11]. US surveys have reported that 5 of every 1000 children aged between 11 and 18 years are diagnosed with Hashimoto thyroiditis every year [11]. Not only an increase in the annual frequency of AIT between 1975 and 2005 (+ 350% after 1995) but also progressive decreases in both the age at presentation and the female-to-male (F/M) ratio were reported in a large Italian study [12].

Autoimmune thyroiditis is responsible for approximately 55–65% of all paediatric euthyroid goitres [13, 14]. It is considered a premalignant lesion with an increased prevalence in cancer [15–17].

The sonographic appearance of lymphocytic thyroiditis varies, likely reflecting the phase and severity of the disease process [12]. In our previous work, we presented five ultrasonographic variants of autoimmune thyroiditis in children (in decreasing frequency): (1) the most common form of diffuse thyroiditis with a hypoechogenic background and normoechogenic parenchyma, (2) diffuse thyroiditis with an irregular background, (3) a nodular variant with a normoechogenic irregular background, (4) micronodulations, and (5) a variant with a diffuse hypoechogenic background [18]. We also reported that patients with the nodular AIT variant with a normoechogenic irregular background of the thyroid gland are at risk of developing PTC and should be followed up with regular neck ultrasound (US) assessments [18].

In the general paediatric population, the incidence of thyroid nodules is 1–18% [19]. In AIT patients, the incidence of thyroid nodules is 13–30% [18–20]. The co-existence of AIT and PTC ranges between 6.3 and 43% depending on the patient population and continues to rise [18, 19, 21–25]. In paediatric patients with PTC, Niedziela et al. found that the prevalence of autoimmune thyroiditis increased tenfold between 1996 and 2000 and 2001–2015 [26]. Increases in both the PTC incidence and the occurrence of PTC with AIT were noted in our centre [14, 18]. Thirteen patients with PTC (30.8% with AIT) between 2000 and 2010 and 20 patients with PTC (55% with AIT) between 2010 and 2017 were treated in our centre in Krakow, Lesser Poland [14].

According to the current paediatric guidelines, neck US in children with autoimmune thyroid disease should be performed at least every 12 months [2]. Since thyroid US is a sensitive and noninvasive procedure, it was incorporated into our institutional follow-up protocol for children with thyroid disorders. Access to ultrasound in our outpatient endocrine clinic enables long-term monitoring of patients in thyroid cancer risk groups, including patients with the normoechogenic AIT variant.

The present study expands upon the previous works from our centre with new observations from ultrasound monitoring of a selected group of AIT patients [14, 18]. The aim of this study was to present the outcomes of ultrasound (US) follow-ups in children with autoimmune thyroid disease who did not have a thyroid nodule on admission but developed PTC and to characterize the parenchymal changes in the thyroid gland prior to the development of PTC that may be useful in identifying a PTC risk group among paediatric patients with AIT.

## Methods

A retrospective chart and thyroid US scan review of 327 patients (245 females) diagnosed with autoimmune thyroiditis (AIT) between January 2015 and December 2017 was performed. The mean age of the patients was 13.1 years (range 3–19 years). The patients were subdivided according to AIT variants (as described before by our group [18]): (a) diffuse thyroiditis with a hypoechogenic background (114/327, 34.9%), (b) diffuse thyroiditis with an irregular background (98/327, 29.9%), (c) micronodulations (65/327, 19.9%), (d) a nodular AIT variant with a normoechogenic background (40/327, 12.2%), and (e) a variant with a diffuse hypoechogenic background (10/327, 3.1%).

The nodular AIT variant with a normoechogenic background was identified in 12.2% (40/327) of the whole group. In 29 patients (29/40, 72.5%) with the nodular AIT variant with a normoechogenic background, fine-needle aspiration biopsy (FNAB) results were benign and corresponded to colloid cysts (3/29, 10.3%), ectopic thymic tissue (2/29, 6.9%), and focal AIT (24/29, 82.8%). Eleven patients with the nodular AIT variant with a normoechogenic background were diagnosed with papillary thyroid carcinoma (nine females, mean age 15.3 years (range 11–19 years); two males aged 11 and 13 years). Patients with PTC constituted 27.5% of the nodular AIT variant cases and 3.4% of the whole group.

In 5 of 11 patients, the suspicious nodule that was later confirmed to be PTC was detected on the initial US at presentation. For the remaining six females (6/11) who developed PTC during the follow-up at the endocrine outpatient department, we retrospectively analysed their US thyroid scans, performed between 2009 and 2017, showing parenchymal changes in the thyroid gland prior to the development of PTC, and these patients were selected for analysis in this study.

The analysis included the reason for referral to the endocrinologist, age at AIT and PTC diagnosis, thyroid status, levels of autoantibodies (TPOAb—thyroperoxidase antibody and TgAb—thyroglobulin antibody), US features of the thyroid gland at presentation (with the date and age of the patient) prior to PTC diagnosis, and US features at the initial detection of the nodule (with the date and age of patient).

AIT was confirmed in six patients based on clinical features (presence of goitre, firm consistency of the thyroid gland), hormonal features (hypothyroidism or subclinical hypothyroidism), and increased TPOAb and/or TgAb levels. Compensated (or subclinical) hypothyroidism was diagnosed if the TSH level was above the upper normal range (*n*: 0.4–4.0 µIU/ml) and fT4 was close to the low normal range (*n*: 10–25 pmol/l). Ultrasonography of the thyroid gland was performed to determine the AIT variant.

US of the thyroid gland was performed at the University Children’s Hospital by board US-certified doctors (DJ, PS, and ŁW). Thyroid US was performed using a high-resolution Voluson 730 GE Medical System (8–12-MHz linear transducer), Philips Epiq5 (L12-5 linear transducer), Philips iE22 (L11-3 linear transducer), and Logiq P6, GE Healthcare (11L linear transducer). The US examination was performed in the transversal and longitudinal planes. Scans indicating background parenchymal echogenicity were reviewed retrospectively. Normal thyroid parenchyma (normoechogenic background) was defined as demonstrating homogenous echogenicity and relative hyperechogenicity compared with the adjacent sternohyoid, sternothyroid, omohyoid, and sternocleidomastoid muscles as described previously [18]. Abnormal parenchymal features of the thyroid gland, including irregular echotexture, micronodularity, and diffuse or focal hypoechogenic lesions and nodules, were also evaluated.

Patients presenting a nodule with suspicious features, such as hypoechogenicity, a hyperechogenic `border` between a nodule and the thyroid parenchyma, poorly defined margins, an irregular shape, microcalcifications, a solid structure, vascularity as detected with Doppler flow, and/or pathologic lymph nodes, were referred to paediatric oncologic surgeons for FNAB. PTC was diagnosed when the FNAB results fulfilled the Bethesda criteria [27]. In patients with PTC, total thyroidectomy with lateral and central lymph node histopathological verification was performed.

Postoperative staging was performed based on the tumour, nodes, and metastases (TNM) system proposed by the American Joint Committee on Cancer [28].

The study was approved by the Institutional Review Board.

## Results

The clinical and hormonal data of the patients are presented in Table 1.

**Table 1:** Clinical and hormonal data of patients

Patient	1	2	3	4	5	6
Age at AIT diagnosis (years)	11 11/12	9 3/12	9 8/12	9 6/12	12 6/12	11 8/12
Age (years) and date (DD-MM-YYYY) of US at presentation	12 23-03-2011	9 3/12 8-08-2015	10 4/12 28-12-2010	9 9/12 27-12-2011	15 5/12 10-07-2015	11 8/12 28-05-2009
Thyroid volume (ml)^a^	10 ml (*N* < 9.5)	11 ml (*N* < 5.4 ml)	5.6 ml (*N* < 5.4 ml)	8.2 ml (*N* < 5.4 ml)	17.7 ml (*N* < 10.9)	21.7 (*N* < 9.5)
TSH at AIT diagnosis (µIU/ml *N*: 0.4–4.0)	5.01	6.6	4.8	8	18.2	9.2
fT4 at AIT diagnosis (pmol/l *N*: 10–25)	11.2	11.1	12.1	10.7	9.7	10.3
TPOAb at AIT diagnosis (IU/ml *N* < 30)	1271.7	10.9	> 1300	226.6	> 1000	> 9000
TgAb at AIT diagnosis (U/ml *N* < 30)	–	195	154.3	–	–	> 1000
Age (years) and date (DD-MM-YYYY) of US at first detection of the nodule	16 8/12 16-11-2015	11 15-05-2017	14 10/12 06-06-2015	13 6/12 09-09-2015	16 9/12 30-11-2016	18 5/12 11-02-2016
Age at PTC diagnosis (years)	16 11/12	11	14 10/12	13 6/12	17 5/12	19
Thyroid volume at PTC diagnosis (ml)	16 ml (*N* < 18 ml)	14.9 ml (*N* < 9.5 ml)	6.4 ml (*N* < 16.1)	11.8 (*N* < 13.1 ml)	6.0 ml (*N* < 18 ml)	9.3 ml (*N* < 18 ml)
Nodule size on US scan (mm)	10.8 × 10.4 × 16.4	6.5 × 4.7 × 6.7	7 × 5 × 6	8 × 8 × 9	6.6 × 9.8 × 4.1	12.2 × 7.4 × 8.9
TSH at PTC dgn (µIU/ml *N*: 0.4–4.0)	0.7	7	2.9	1.5	3.5	0.7
fT4 at PTC diagnosis (pmol/l *N*:10–25)	16.8	9.2	16.7	19.3	15.3	19.8
TPOAb at PTC dgn (IU/ml *N* < 30)	–	167.7	925.9	126.2	1920	245.2
TgAb at PTC dgn (U/ml *N* < 30)	–	93.4	21.1	154.3	467.5	404.2
Time to PTC detection since referral (years)	5	1 9/12	4 10/12	4	4 11/12	7 4/12
Time to PTC detection since last nodule-free US scan (years)	4 8/12	10/12	6/12	3 9/12	1 4/12	6 9/12
AACE/ACE/AME risk group	Class 3	Class 3	Class 3	Class 3	Class 3	Class 3
TNM	pT1aN0M0	pT1aN0M0	pT1aN0M0	pT1aN1aM0	pT1aN0M0	pT1aN0M0
ATA risk group	I	I	I	II	I	I
PTC variant	Classic	Follicular	Classic, solid, follicular	Classic, follicular	Classic	Classic

ATA (The American Thyroid Association) pediatric risk group: I—low risk, II— intermediate risk, and III—high risk [39]

AACE/ACE/AME (The American Association of Clinical Endocrinologists, American College of Endocrinology and Associazione Medici Endocrinologi) US Classification System: Class 1—Low-risk thyroid lesion; Class 2—Intermediate-risk thyroid lesion; Class 3—High-risk thyroid lesion [40]

*TPOAb* thyroperoxidase antibody, *TgAb* thyroglobulin antibody, *AIT* autoimmune thyroiditis, *PTC* papillary thyroid carcinoma, *TNM* tumour, nodes, and metastases system

^a^Thyroid volume references [41, 42]

**Fig. 1:**
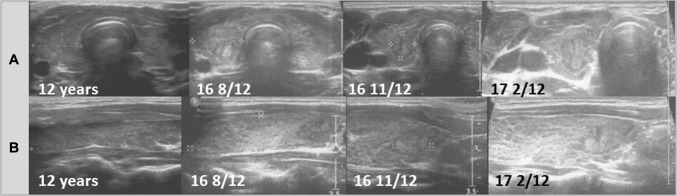
Patient 1. Transversal (**a**) and longitudinal (**b**) scans of the hypoechogenic thyroid gland revealed formation of the nodule 5 years since referral and 4 8/12 years since last ultrasound assessment. The growth of the nodule was fast from 8.1 × 9.5 × 13.1 mm to 10.8 × 10.4 × 16.4 mm in 6 months (82% increase). During follow-up, the echogenicity of the thyroid gland increased

**Fig. 2:**
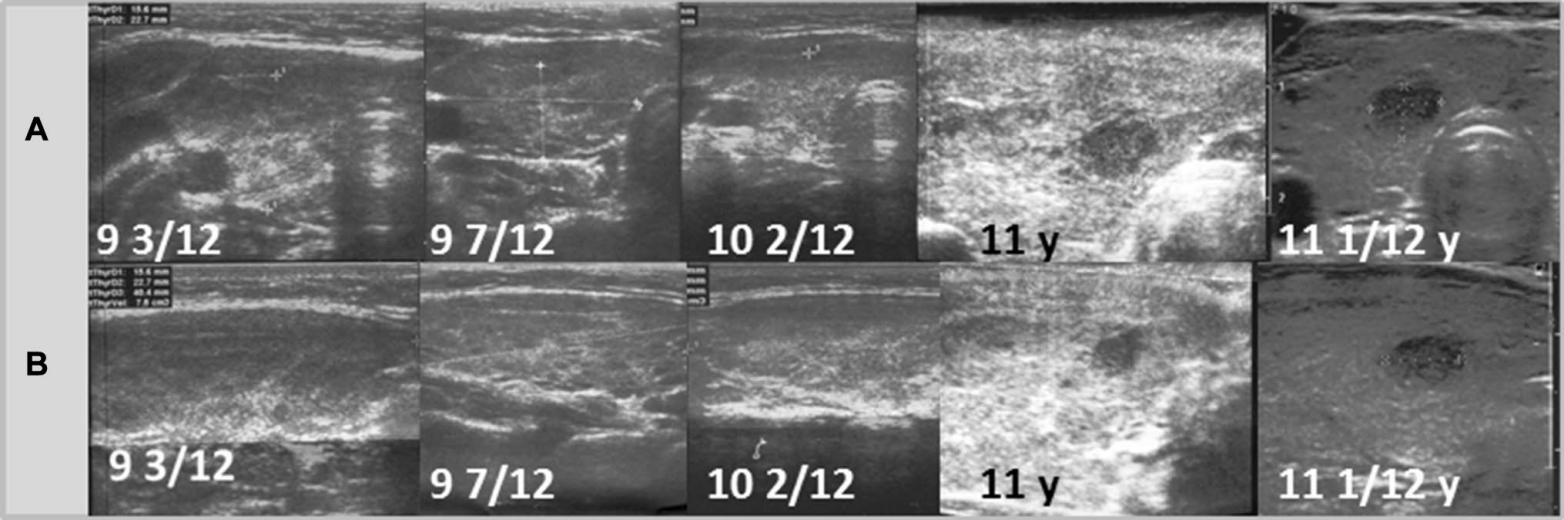
Patient 2. Transversal (**a**) and longitudinal (**b**) scans of the hypoechogenic thyroid gland revealed the formation of the nodule 1 9/12 years since referral and 10/12 months since last ultrasound assessment. The growth of the nodule was fast from 5.4 × 4.7 × 4.2 mm to 6.5 × 4.7 × 6.7 mm in 1 month (92% increase). During follow-up, the echogenicity of the thyroid gland increased

**Fig. 3:**
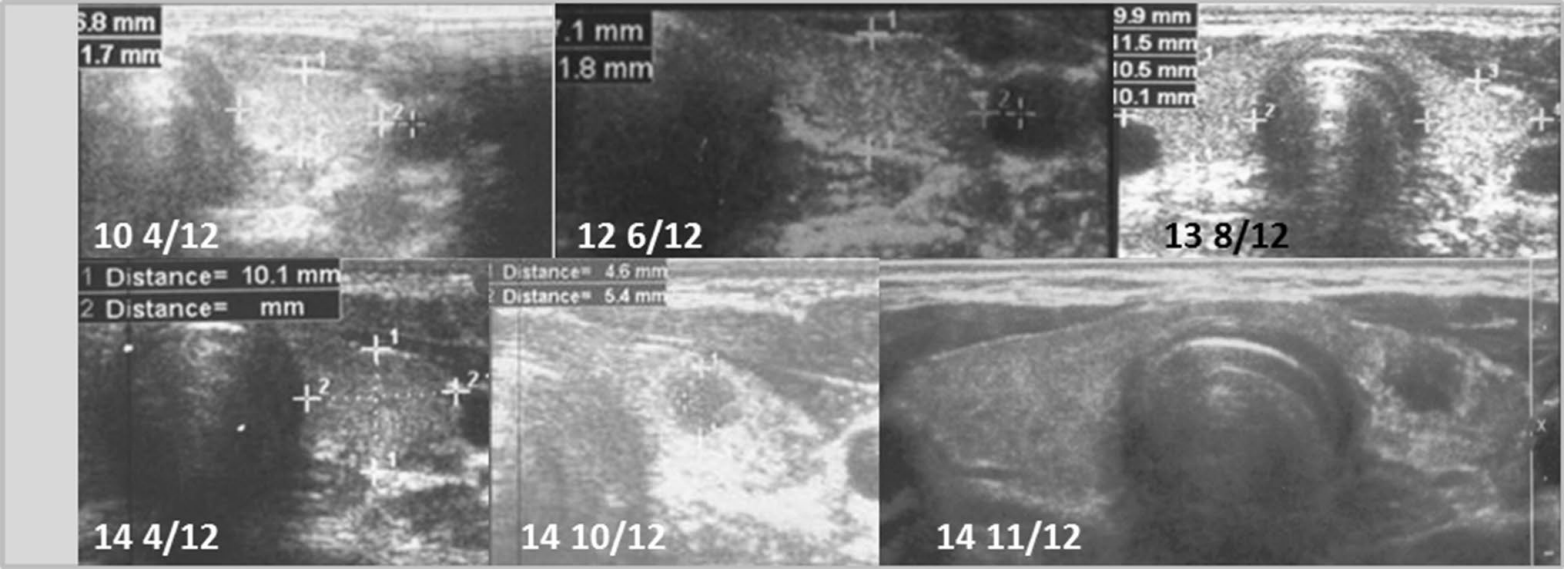
Patient 3. Transversal scans of the normoechogenic thyroid gland revealed the formation of the nodule 4 10/12 years since referral and 6 months after last ultrasound assessment. The growth of the nodule was fast from 5.4 × 4.6 × 4.6 mm to 7 × 5 × 6 mm (84% increase) in 1 month. The nodule was surrounded by a hyperechogenic border

**Fig. 4:**
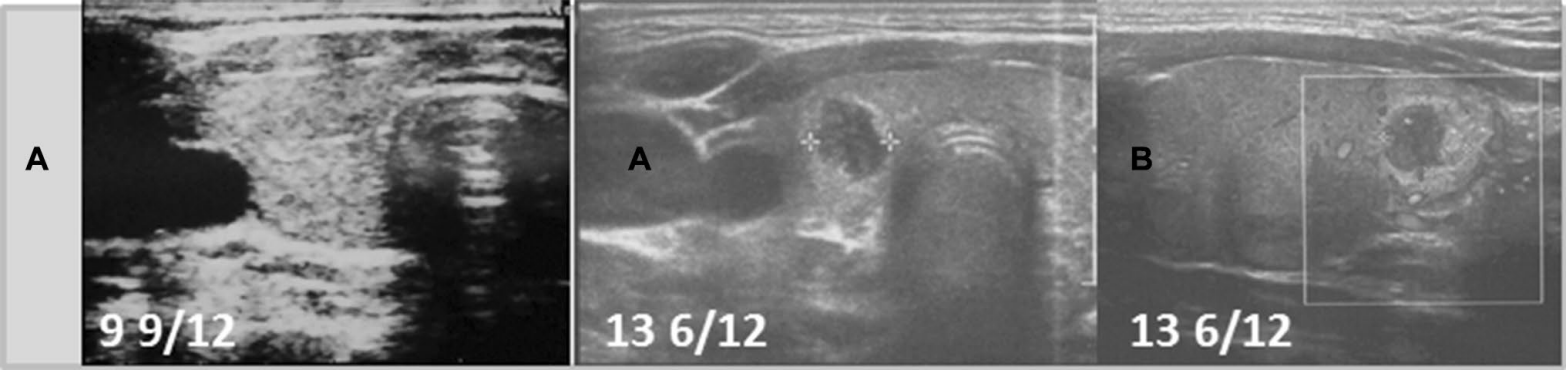
Patient 4. Transversal (**a**) and longitudinal (**b**) scans of the normoechogenic thyroid gland revealed the formation of the nodule 4 years since referral and 3 9/12 months since last ultrasound assessment. The nodule was surrounded by a hyperechogenic border. During follow-up, the echogenicity of the thyroid gland increased

**Fig. 5:**
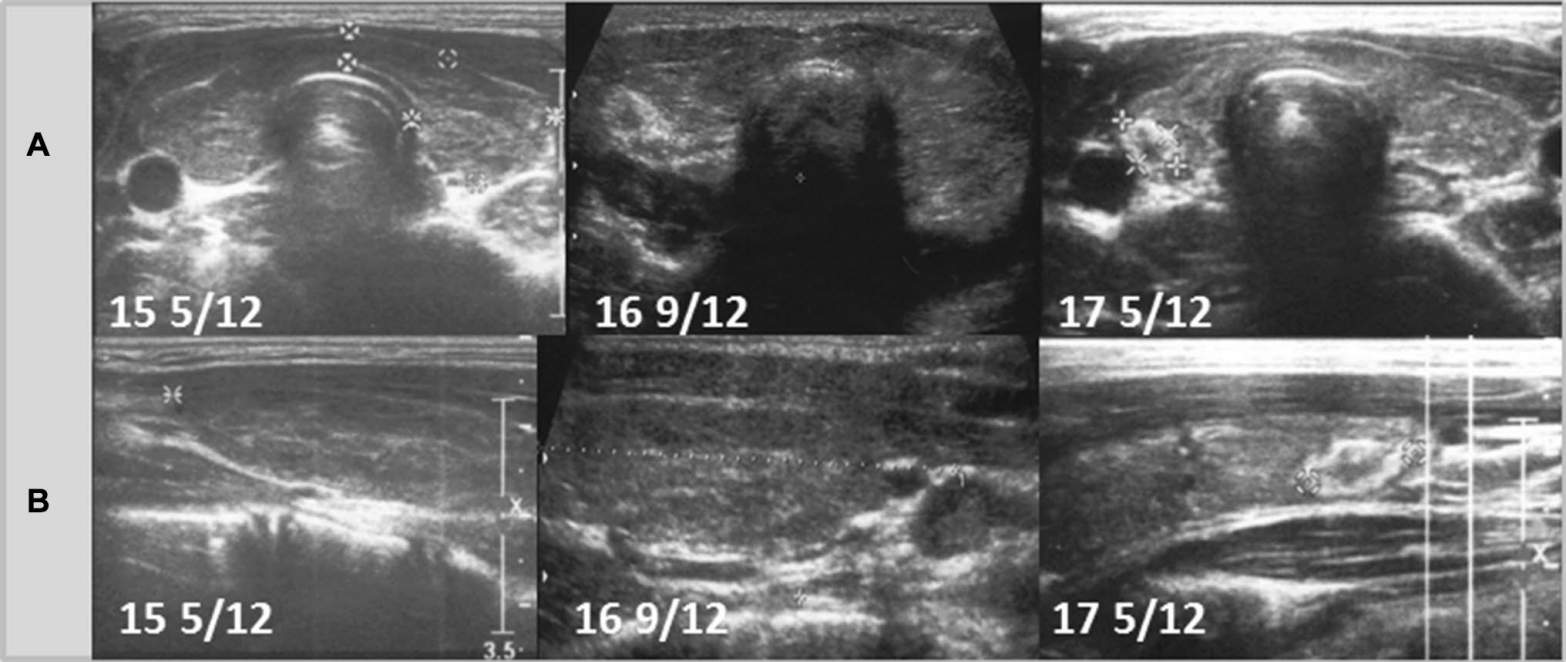
Patient 5. Transversal (**a**) and longitudinal (**b**) scans of the hypoechogenic thyroid gland revealed the formation of the nodule 4 11/12 years since referral and 1 4/12 year since last ultrasound assessment. The growth of the nodule was fast from 6.5 × 5.5 × 3.2 mm to 6.6 × 9.8 × 4.1 mm in 8 months (132% increase). The nodule was surrounded by a hyperechogenic border. During follow-up, the echogenicity of the thyroid gland increased

**Fig. 6:**
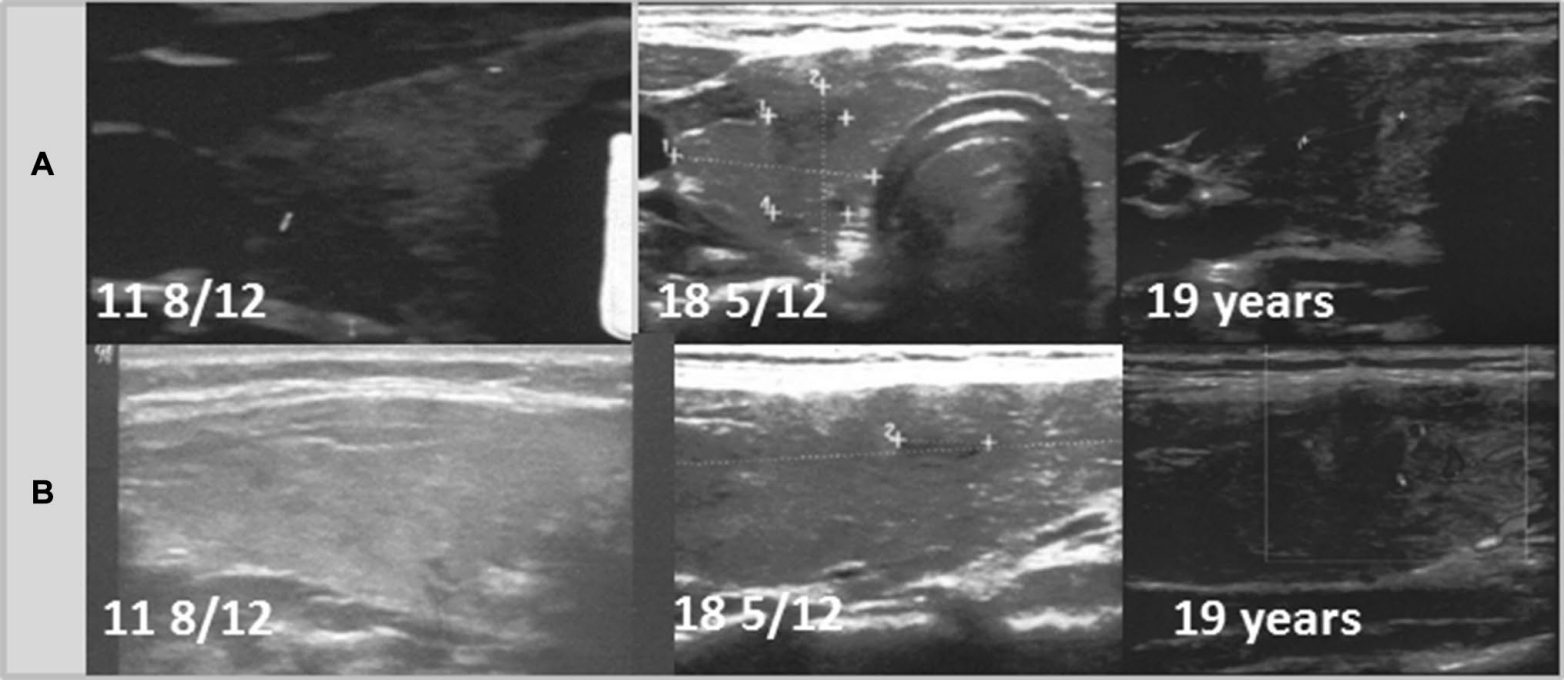
Patient 6. Transversal (**a**) and longitudinal (**b**) scans of the normoechogenic thyroid gland revealed formation of the nodule 6 9/12 years since referral and since last ultrasound assessment. The growth of the nodule was fast from 6.3 × 7.3 × 4.2 mm to 12.2 × 7.4 × 8.9 mm in 7 months (316% increase). The nodule was surrounded by a hyperechogenic border

Ultrasound follow-ups with transversal and longitudinal scans of the thyroid glands of all patients are presented in Figs. 1, 2, 3, 4, 5, and 6.

All patients were referred for endocrine evaluation because of an enlarged thyroid gland. Thyroid palpation revealed diffuse firm goitre in all cases.

On admission, the US evaluation revealed an enlarged normoechogenic thyroid gland in three patients and a hypoechogenic thyroid gland with fibrosis as indicated by irregular, chaotic hyperechogenic layers in three patients. The median thyroid volume was 10.5 ml (range 5.6–21.7 ml, the reference thyroid volume data for age and gender are presented in Table 1 and separately for individual patients). At the time of the initial US, no thyroid nodules were identified in the study group.

One patient was diagnosed with hypothyroidism (patient 5), and five patients were diagnosed with subclinical hypothyroidism. Because of the presence of a goitre and abnormal thyroid function, all patients received therapy with levothyroxine. TPOAb levels were increased in five patients (1, 3–6). In patient 2, the TPOAb level was within the normal range, but the TgAb level was increased, thus confirming autoimmunity (Table 1).

Ultrasound monitoring revealed increasing echogenicity of the thyroid parenchyma in relation to the adjacent sternothyroid, sternohyoid, sternocleidomastoid, and omohyoid muscles in all patients during the follow-up. Finally, in all patients, malignant nodules developed in the thyroid gland with a normoechogenic background without typical ultrasound AIT features such as diffuse hypoechogenicity or micronodulations as demonstrated in our previous study [18]. Papillary thyroid carcinoma developed in a mean time of 4.6 years (1 9/12–7 4/12 years) since referral to the outpatient thyroid clinic and 2.9 years (6/12–6 9/12) since the last nodule-free US thyroid scan (Figs. 1, 2, 3, 4, 5, 6). The median maximum nodule size on US was 9.4 mm (range: 6.7–16.4 mm) and the growth rate of the nodules was fast (Figs. 1, 2, 3, 4, 5, and 6). The volume of the nodule increased by 82% at 6 months in Patient 1, 92% at 1 month in Patient 2, 84% at 1 month in Patient 3, 132% at 8 months in Patient 5, and 316% at 7 months in Patient 6 during observation in the outpatient clinic before FNAB was performed.

In five patients, the nodules were hypoechogenic. In four of these patients, the nodules were surrounded by a hyperechogenic ‘margin’, and in one patient, the nodule had mixed echogenicity and an irregular lobulated border. No microcalcifications were observed in the nodules. The vascularization of the nodules was centralized and increased in relation to the surrounding thyroid parenchyma.

Apart from one non-compliant patient who stopped levothyroxine treatment for 1 year (Patient 2), in all cases, decreases in goitre volume were observed at the time of PTC diagnosis from a median volume of 10 ml (5.6–21.7 ml) on US at presentation to a median of 9.3 ml (6–16 ml). The median TSH levels decreased from 8 µIU/ml (5.01–18.2) to 1.5 µIU/ml (0.7–7.0).

After FNAB confirmation according to the Bethesda criteria (category V-suspicious for malignancy) [27], total thyroidectomy with central and lateral lymph node dissection was performed in all patients.

TNM classification revealed pT1aN0M0 in 5/6 patients, and these patients did not receive I^131^ therapy. Only Patient 4 was classified as pT1aN1aM0 with a nodule < 10 mm and received radionuclide therapy.

The classic PTC variant was found in three patients, the follicular PTC variant was found in one patient, and mixed variants were found in two patients: classic/solid/follicular and classic/follicular (Table 1).

After surgery, the outcomes of therapy were favourable. No complications after thyroidectomy were noted. All patients are currently followed up at the Institute of Oncology and are in remission. Tg and TgAb levels are negative in all cases.

## Discussion

To our knowledge, this is the first report of ultrasound presentations of parenchymal changes in the thyroid gland in young patients with autoimmune thyroiditis evaluated sonographically prior to the development of histologically confirmed papillary thyroid carcinoma.

In the overall group of 327 AIT patients, the distribution of sonographic AIT variants was similar to our observation in the previous work [18]. A total of 87.8% of the patients presented typical US variants of AIT (diffuse thyroiditis with a hypoechogenic background, diffuse thyroiditis with an irregular background, micronodulations, or a diffuse hypoechogenic background) and changes were not observed in the parenchyma or the nodules in this group of patients during the follow-up [18].

The nodular AIT variant with a normoechogenic background was identified in 12.2% of the whole group. Eleven patients (female predominance) presenting this variant were diagnosed with papillary thyroid carcinoma. Patients with PTC constituted 27.5% of the nodular AIT variant cases and 3.4% of the whole group; these results are similar to those of other paediatric series [19, 20].

In six females who did not have a thyroid nodule on admission but who developed PTC, we have been able to retrospectively analyse US thyroid scans presenting parenchymal changes in the thyroid gland prior to the development of PTC and these patients were chosen for presentation in this study.

PTC was detected early via US in our study before the nodule was clinically apparent. Four of six confirmed cancer cases were associated with thyroid nodules < 1 cm, which are difficult to detect by manual palpation alone. The most common sonographic presentation of a malignant lesion in our group was a solid hypoechoic nodule with an irregular hyperechoic margin (histopathologically identified as fibrosis and lymphocytic infiltration) and without microcalcifications. The detected nodules showed fast growth rates over time, with volumes that doubled or even tripled within 6–8 months of observation before FNAB was performed. In one patient with a nodule diameter less than 10 mm, metastases to several lymph nodes were detected and this patient received radioactive iodine therapy (RAI).

Our results suggest that the use of US can increase the detection rate of thyroid malignancies in patients with the normoechogenic AIT variant who seem to be at a higher risk of cancer than the overall population of children with autoimmune thyroiditis [18]. This higher risk may justify the need for closer monitoring, especially considering the rapid growth rate of malignant nodules in young patients. Unlike adults, children with papillary thyroid cancer may present with more advanced disease and have higher rates of local recurrence and distant metastases, even though their prognosis is favourable, with overall 10-year survival rates of 80–95% [29]. Children have a longer posttreatment life expectancy and, therefore, more time for recurrence or potential treatment effects to manifest [1]. Recent studies have confirmed that radioactive iodine (RAI) ablation is associated with an increased risk for the development of additional malignancies as well as an increase in overall mortality for patients with DTC [30]. Frequent US follow-up examinations enable the early detection of PTC, radical surgery, and avoidance of RAI therapy. In our study, five of six patients did not receive radionuclide therapy, which will probably impact their future quality of life considering the long-term side effects associated with RAI [30]. We are convinced that certain sonographic features of the thyroid gland observed in our follow-up study may facilitate the early detection of malignancies.

An ongoing debate in the literature is whether the nodular variant of AIT with a normoechogenic background indicating the presence of residual thyroid tissue identified in patients with PTC is a different type of disease compared to diffuse AIT and whether autoimmune thyroiditis is secondary to cancer in this group of patients [18, 31–34]. As presented in our previous study, patients with typical variants of diffuse AIT more often developed abnormalities of thyroid function (overt hypothyroidisms or hyperthyroidism) compared to patients with the nodular AIT variant with a normoechogenic background [18]. The decreased echogenicity of the parenchyma in diffuse AIT has been shown to be related to lymphocyte infiltration and correlated with hypothyroidism [31, 34]. A lower incidence of hypothyroidism in patients with nodular AIT and normal background thyroid parenchyma was also observed in other studies [33, 34]. Our findings are consistent with those of Oppenheimer et al., indicating that at least two distinct patterns of AIT exist on thyroid sonography: diffuse AIT and nodular AIT [31]. As hypothesized by Paparodis et al. and Imam et al., in patients with euthyroid/functional AIT and low titres of TPOAb, a different immune disorder that does not completely destroy the thyroid gland may be present, or cancer may actively participate in regulating immunity or autoimmunity (cancer immunoediting) [33, 35]. As suggested by Ehlers and Schott, the reason for the induced antitumour immune response (with increased but low TPOAb or TgAb levels in serum) may be the presence of undiagnosed papillary thyroid microcarcinomas [32]. Consistent with this observation, Paparodis et al. reported an association between AIT duration and PTC development after finding that a shorter AIT duration was associated with PTC development, while a longer duration was not [33]. Interestingly, Italian authors reported that the annual increase in the AIT incidence preceded the annual increase in the PTC incidence, and suggested that environmental influences may have favoured both thyroid autoimmune disease and PTC-oriented thyroid oncogenesis [36]. The new information reported in our study is consistent with the hypothesis that autoimmune thyroiditis may be secondary to thyroid oncogenesis as half of our patients exhibited normoechogenic thyroid parenchyma prior to the development of PTC. In our study group, three girls had a normoechogenic thyroid gland at presentation, and whether these findings reflected the early stage of AIT or a normal thyroid gland was questionable considering that up to 20% of the population has positive thyroid autoantibodies without any thyroid disease [31]. AIT was confirmed in our patients by the presence of goitre, subclinical hypothyroidism, and autoantibodies to thyroglobulin and thyroid peroxidase. Follow-up scans did not reveal typical sonographic AIT features (micronodularity or decreased echogenicity with visible hyperechogenic septations). However, during the follow-up, we detected a solitary hypoechogenic nodule that was later confirmed to be PTC. Therefore, we suggest that not only nodular AIT but also AIT with a normoechogenic background on sonography at presentation (with no nodules) may be an entirely different clinical entity with an increased PTC risk, which warrants further research in the paediatric population [18, 31, 33]. Our study also found that during the follow-up of hypoechogenic thyroid glands with irregular hyperechogenic layers, an increase in echogenicity due to parenchymal changes was evident, probably due to fibrosis and follicular destruction in the thyroid gland during the disease process prior to detection of a nodule [37, 38].

In our AIT cohort, the incorporation of thyroid US follow-ups enabled relatively early detection of thyroid malignancies that were not clinically apparent over a mean of 4.6 years since referral and AIT diagnosis. The data presented here are consistent with those of the Italian study by Rizzo et al., demonstrating that the lag time between AIT diagnosis and PTC detection was approximately 5 years, which is very similar to the average value reported in our paediatric study [36]. In addition, a novel and practical finding of our study is that the shortest lag time from the last nodule-free scan to the detection of a suspicious nodule was 6 months.

Another point of interest in our study is the observation that although all patients received levothyroxine treatment, except for one patient, and exhibited decreased goitre volumes and TSH levels, l-thyroxine replacement therapy failed to provide any protection from nodule development. This finding may support the hypothesis that thyroid oncogenesis may precede autoimmunity; however, further research on larger series of patients is required to confirm these observations.

Several limitations exist in our study. Due to the low incidence of PTC in young patients, our sample was small; therefore, the study had insufficient power to establish statistical significance. Nevertheless, we propose that sonographic follow-up assessments warrant further exploration as a strategy to determine PTC susceptibility in the paediatric population.

In accordance with the ATA and Polish Guidelines, children with AIT should undergo US evaluation annually. We are convinced, however, that children with a normoechogenic background of the thyroid gland or a hypoechogenic background with fibrosis appearing as irregular hyperechogenic layers should be considered to have a higher risk of cancer and should be followed more closely with more frequent ultrasound evaluations [2, 39].
